# The clinical approach to child and adolescent patients with lipodystrophy: a series of international case discussions

**DOI:** 10.3389/fendo.2025.1597053

**Published:** 2025-08-06

**Authors:** Rebecca J. Brown, Baris Akinci, Saif Al Yaarubi, Elise Bismuth, Marco Cappa, Asma Deeb, Clemens Kamrath, Carla Musso, Nivedita Patni, Flavia Prodam, Rachel Williams, Martin Wabitsch

**Affiliations:** ^1^ National Institute of Diabetes and Digestive and Kidney Diseases, National Institutes of Health, Bethesda, MD, United States; ^2^ Department of Technological Research, Izmir Biomedicine and Genome Center, Dokuz Eylül University, Izmir, Türkiye; ^3^ Oman Medical Specialty Board, Muscat, Oman; ^4^ Department of Pediatric Endocrinology, Assistance Publique-Hôpitaux de Paris, Robert Debré Hospital, Paris, France; ^5^ Research Unit for Innovative Therapies in Endocrinopathies, Bambino Gesù Children’s Hospital, Istituto di Ricovero e Cura a Carattere Scientifico (IRCCS), Rome, Italy; ^6^ Division of Paediatric Endocrine, Sheikh Shakhbout Medical City and College of Medicine and Health Sciences, Khalifa University, Abu Dhabi, United Arab Emirates; ^7^ Centre of Child and Adolescent Medicine, Department of General Pediatrics and Neonatology, University Hospital Freiburg, Freiburg, Germany; ^8^ Hospital Universitario Fundación Favaloro, Ciudad Autónoma de Buenos Aires, Argentina; ^9^ Division of Pediatric Endocrinology, Department of Pediatrics, University of Texas (UT) Southwestern Medical Center, Dallas, TX, United States; ^10^ Unit of Endocrinology, Department of Health Sciences, University of Eastern Piedmont, Novara, Italy; ^11^ Cambridge University Hospitals, National Health Service (NHS) Foundation Trust, Cambridge, United Kingdom; ^12^ Nottingham Children’s Hospital, Nottingham, United Kingdom; ^13^ German Center for Child and Adolescent Health (DZKJ), Ulm Site, Division of Pediatric Endocrinology and Diabetes, Department of Pediatrics and Adolescent Medicine, University Medical Center, Ulm, Germany

**Keywords:** acquired generalized lipodystrophy, acquired partial lipodystrophy, comorbidities, congenital generalized lipodystrophy, familial partial lipodystrophy, pediatric, metreleptin, adolescent

## Abstract

**Introduction:**

Lipodystrophy syndromes comprise a group of rare endocrine disorders characterized by the generalized or partial loss of adipose tissue. Affected individuals frequently display absolute or relative reductions in leptin, a key adipokine regulator of hunger-satiety signaling, and are predisposed to a range of metabolic and end-organ complications, often from a young age. The presentation and severity of lipodystrophy syndromes is largely dependent on the extent of adipose tissue loss while comorbidities often deteriorate with age. In this regard, optimizing care for children and adolescents with lipodystrophy syndromes is a pivotal step in supporting them into adulthood. To assist clinicians with limited experience of managing young patients with lipodystrophy syndromes, we describe our clinical approach to a series of pediatric patients with these rare diseases.

**Methods:**

The clinical history, diagnosis, disease management and follow-up care of 10 international pediatric patients with lipodystrophy syndromes are presented. Teaching points from each case study are also provided. Most of these cases are based on patients from our clinics with certain details changed to protect privacy. Others represent hypothetical scenarios based on our clinical experience supported by review of the medical literature and are included here for educational purposes.

**Results:**

Our patients illustrate the broad phenotypic spectrum of lipodystrophy syndromes that can manifest early in life. We highlight the importance of timely and accurate diagnosis in guiding early disease management strategies to help reduce the risk of comorbidities. The challenges faced by clinicians managing pediatric patients with lipodystrophy syndromes and how these challenges may differ from adult patients are also explored.

**Discussion:**

The cases presented in this manuscript may assist clinical teams to promptly diagnose and holistically manage young patients with lipodystrophy syndromes and help optimize clinical outcomes as they transition to adult care.

## 1 Introduction

Lipodystrophy syndromes are a heterogeneous group of rare diseases characterized by irreversible adipose tissue loss that can affect either the whole body (generalized lipodystrophy, GL) or specific areas (partial lipodystrophy, PL) (1–3). Generalized and partial lipodystrophies are further categorized by etiology (i.e., genetic or acquired), giving rise to four main types: congenital generalized lipodystrophy (CGL, or Berardinelli-Seip syndrome), familial partial lipodystrophy (FPLD), acquired generalized lipodystrophy (AGL, or Lawrence syndrome) and acquired partial lipodystrophy (APL, or Barraquer-Simons syndrome) (1, 2, 4–10) (**Table 1**).

**Table 1:** Classification and clinical features of the main lipodystrophy syndrome types.

Type	Generalized lipodystrophy		Partial lipodystrophy	
	CGL	AGL	FPLD	APL
Distribution of adipose tissue loss	• Almost complete lack of adipose tissue	• Almost complete lack of adipose tissue	• Partial adipose tissue loss primarily affecting the upper and lower extremities and sometimes the trunk • Fat may accumulate (especially in females) on the face and neck (in FPLD2) or in the intra-abdominal region often leading to truncal obesity (FPLD1)	• Adipose tissue loss follows cranio-caudal trend • Adipose tissue loss typically affects the face, neck and upper part of the body • Adipose tissue is often preserved or increased in the lower body (especially in females)
Age of adipose tissue loss onset	• Adipose loss typically evident at birth or can occur during infancy or early childhood	• Progressive loss of adipose tissue occurring over a period that can range from a few weeks to years • Adipose tissue loss can occur at any stage in life but typically occurs during childhood and adolescence	• Fat loss usually becomes prominent during puberty	• Adipose tissue loss usually begins in childhood or adolescence and may progress slowly over years
Etiology	Subtypes (gene) • CGL1 (*AGPAT2*) *• CGL2 (BSCL2*) • CGL3 (*CAV1*) • CGL4 (*CAVIN1*/*PTRF*) All subtypes listed above are associated with autosomal recessive inheritance Other genes associated with the generalized fat loss include *LMNA* variants, biallelic *PPARG* variants, *PCYT1A* and *PLAAT3*	Subtypes • Autoimmune • Panniculitis-associated • Idiopathic	Subtype (gene, mode of inheritance) • FPLD1 or Köbberling type (unknown, possibly polygenic) • FPLD2 or Dunnigan type (*LMNA*, autosomal dominant) • FPLD3 (*PPARG*, autosomal dominant) • FPLD4 (*PLIN1*, autosomal dominant) • FPLD5 (*CIDEC*, autosomal recessive) • FPLD6 (*LIPE*, autosomal recessive) Other genes associated with partial fat loss include *MFN2*, *AKT2* and *NOTCH3*	Subtypes • Autoimmune • MPGN-associated • Idiopathic
Leptin levels	• Low or undetectable	• Low or undetectable	• May be low or within normal range	• May be low or within normal range
Notable clinical features	• Accelerated linear growth and advanced bone age • Severe metabolic/organ system comorbidities are common	• Metabolic/organ system comorbidities are common and are generally severe	• Metabolic complications are common and can be severe	• Metabolic abnormalities are less common (vs of lipodystrophy types) and can vary in severity

Not all features may be present in all patients with lipodystrophy and the frequencies at which features occur can vary among lipodystrophy types (1–3, 32, 37–39, 43, 46, 103). AGL, acquired generalized lipodystrophy, APL, acquired partial lipodystrophy; CGL, congenital generalized lipodystrophy; FPLD, familial partial lipodystrophy; MPGN, membranoproliferative glomerulonephritis.

The prevalence of lipodystrophy syndromes remains unclear. Early work estimated the prevalence of genetic lipodystrophies at less than 1 case per million (11). Subsequent interrogation of electronic medical records yielded a prevalence for all lipodystrophy types at 1.3–4.7 cases per million, with estimates of 0.23 and 2.84 per million for GL and PL, respectively (12). Recent genetic modeling combined with data from two ongoing international registries estimated the prevalence of FPLD at 19–30 cases per million (13). It is recognized, however, that many individuals with lipodystrophy syndromes are undiagnosed, especially those with PL, leading to a widespread underestimation of the true prevalence (13, 14).

**Figure 1:**
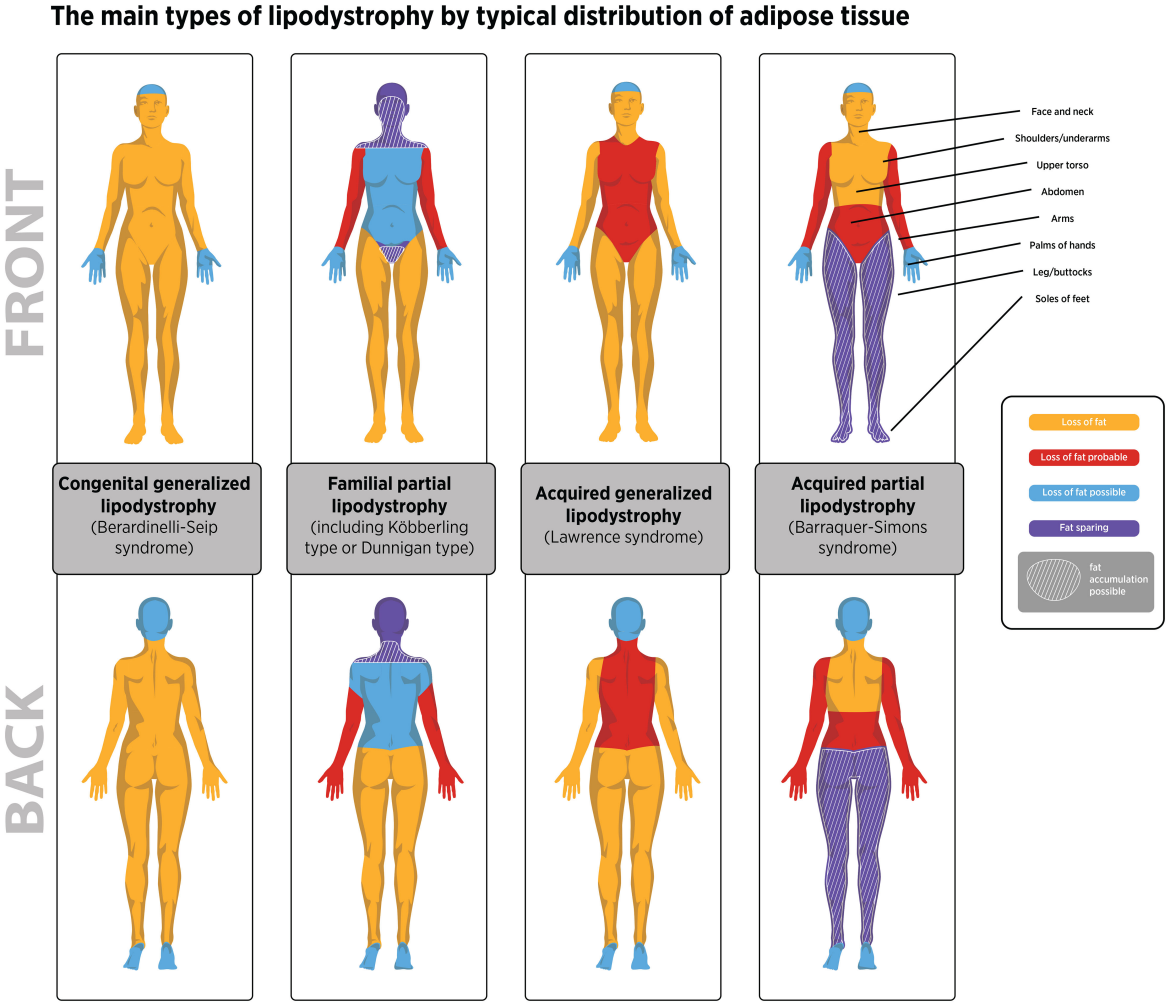
The distribution of adipose tissue loss observed for the four main lipodystrophy types. Illustrative purposes only. Adipose loss patterns can vary between individuals with lipodystrophy based on published information (1–3). In lipodystrophy fat distribution is dependent on the lipodystrophy type presented by the patient and not by biological sex. Areas where fat may accumulate in familial partial lipodystrophy are also shown. Fat may also accumulate in the lower body in some individuals with APL.

Genetic causes of lipodystrophy are predominantly associated with pathogenic variants in genes involved in adipocyte function and development (1, 2, 11). CGL is primarily an autosomal recessive disease characterized by a near-complete lack of body fat, which is recognizable in infancy in most cases (1, 2, 5, 9). At least four CGL subtypes have been described, each linked to variants in four distinct genes (15–24). FPLD is characterized by the selective loss of fat from the limbs and buttocks, which generally begins around puberty and becomes apparent in young adulthood (1, 2, 6, 7). There are at least six FPLD subtypes, most of which show autosomal dominant inheritance (25–36). In acquired lipodystrophies, adipose tissue is normally distributed at birth but is lost at some stage in life (1, 2, 11). The rate of adipose tissue loss in acquired lipodystrophies ranges from months to years, and reduced adiposity is typically evident before or during adolescence (4, 8, 10, 11, 37–39). Although the exact etiology of acquired lipodystrophies is unknown, many cases are associated with underlying autoimmunity (11, 38–42). The patterns of adipose tissue loss and clinical features of the main lipodystrophy subtypes are summarized in **Table 1** and **Figure 1**. Additional details of genes associated with the main types of genetic lipodystrophies are presented in **Table 2**.

**Table 2:** The molecular function of genes associated with genetic lipodystrophies.

Congenital generalized lipodystrophy (CGL)		Familial partial lipodystrophy (FPLD)	
Subtype (gene)	Gene function	Subtype (gene)	Gene function
CGL1 (*AGPAT2*) CGL2 (*BSCL2*) CGL3 (*CAV1*) CGL4 (*CAVIN1*/*PTRF*)	*AGPAT2* is highly expressed in adipose tissue. AGPAT proteins play a role in triglyceride and phospholipid biosynthesis. *BSCL2* encodes seipin, which is thought to play a role in lipid droplet formation and adipocyte differentiation. Caveolin 1 forms a key component of caveolae, (i.e., plasma membrane invaginations abundant on adipocyte membranes) that bind and translocate fatty acids to lipid droplets. PTRF is involved in caveolae biogenesis and regulates the expression of caveolin 1 and 3.	FPLD1 FPLD2 (*LMNA*) FPLD3 (*PPARG*) FPLD4 (*PLIN1*) FPLD5 (*CIDEC*) FPLD6 (*LIPE*)	Unknown genetic cause, possibly polygenic. *LMNA* encodes the lamin A and C nuclear lamina proteins. Mutations in this gene disrupt the interaction between nuclear lamina and chromatic resulting in premature death of adipocytes. PPARG is a transcription factor involved in adipogenesis. Pathogenic variants in the *PPARG* gene may impair adipocyte differentiation. *PLIN1* encodes perilipin 1 encodes a lipid droplet coat protein. Histologically, individuals with FPLD4 have small adipocytes. CIDEC is expressed in the lipid droplets. Pathogenic variants in the *CIDEC* gene may impair the ability of lipid droplets to store fat. *LIPE* encodes hormone sensitive lipase E which is involved in the regulation of lipolysis by adipocytes. Pathogenic variants in the *LIPE* gene may impair lipolysis and induce lipomatosis and partial fat loss.

Data compiled from (2, 11, 30, 32).

Patients with lipodystrophy syndromes present with a range of comorbidities (1, 2, 11, 37, 43). The deficiency of adipose tissue causes ectopic lipid storage (e.g., in the liver and muscles) and predisposes patients to the risk of developing severe metabolic disease (e.g., severe insulin resistance, hypertriglyceridemia, metabolic dysfunction-associated steatotic liver disease) and end-organ damage (e.g., liver, kidney, and cardiovascular disease, and reproductive dysfunction), often from a young age (44–47). Other common clinical features include acanthosis nigricans, polycystic ovarian syndrome (PCOS), eruptive xanthomas (due to severe hypertriglyceridemia), generalized muscularity (in GL) or increased muscularity of the limbs (in PL), prominent veins and umbilical protuberance (in GL) (1–3). Acromegaloid features (in CGL) and Cushingoid features (in FPLD) may also develop in some patients (1–3). Furthermore, patients frequently display absolute or relative reductions in leptin levels, causing impaired hunger-satiety signaling and hyperphagia, and the metabolic complications of leptin deficiency (48–51).

Clinical management of lipodystrophy syndromes aims to improve or prevent the development of comorbidities (1, 52). Lifestyle modifications involving energy-restricted diets and exercise (if not contraindicated) are pivotal. Widely used treatments include glucose- and lipid-lowering therapies, cardiovascular medications, and other agents targeting the complications of the presenting patient (1, 52). Metreleptin, a recombinant form of human leptin, is the only therapy currently approved to treat the metabolic complications of lipodystrophy syndromes (53). Metreleptin was first approved in Japan in 2013 for the treatment of lipodystrophy (54, 55). In the United States, metreleptin is indicated as an adjunct to diet as replacement therapy to treat the complications of leptin deficiency in patients with GL (56). In Europe, Brazil and Canada, metreleptin is approved as an adjunct to diet as replacement therapy to treat the complications of leptin deficiency in patients aged ≥ 2 years with confirmed GL, or in patients aged ≥ 12 years with confirmed PL (with persistent significant metabolic disease, Canada only) for whom standard treatments have not achieved adequate metabolic control (57–60). The current multi-society practice guideline for lipodystrophy syndromes recommends metreleptin (with diet) as a first-line treatment for the metabolic complications of GL, including pediatric patients (1).

The presentation and severity of lipodystrophy syndromes can vary depending on disease subtype and the extent of adipose tissue loss, with comorbidities often worsening with age (43, 44, 47, 61). In this regard, timely and accurate diagnosis of pediatric patients with lipodystrophy syndromes can help inform early disease management strategies that support patients into adulthood (52, 62). To assist clinical teams with limited experience in managing pediatric patients with lipodystrophy syndromes, we describe the clinical approach used by us in a series of international patients with this rare disease. Most of these cases are based on patients from our clinics with certain details changed to protect privacy. Others represent hypothetical scenarios based on our clinical experience supplemented with a cumulative review of the literature and are included here for educational purposes.

## 2 Results

The key clinical features, follow-up outcomes and learnings for each case are summarized in **Supplementary Figure 1**.

### 2.1 Case study 1 – a case of CGL2 with early onset advanced liver disease

#### 2.1.1 Clinical history and diagnostic evaluation

Case 1 describes a 6-year-old female with CGL2 who is clinically managed in North America. She was diagnosed at 5 years of age based on muscular appearance with lack of subcutaneous fat, hypertriglyceridemia, and hepatic steatosis. Her sister had CGL2, an older brother with a similar physical appearance died at age 7 years of unknown cause, and her parents were consanguineous. She had a large appetite with lack of satiety after meals. Physical examination revealed generalized lack of subcutaneous fat, including absence of mechanical fat in the face, palms, and soles. She was tall for her age, with Z scores for height +2.5, weight +2.0, and body mass index (BMI) +1.6. There was acanthosis nigricans affecting the neck, axillae, and groin. Abdominal girth was markedly increased, with liver edge 15 cm below the right costal margin. Fasting laboratory values revealed a normal glucose level (83 mg/dL [4.6 mmol/L]), markedly elevated insulin level (111 μU/mL [771 pmol/L]), and a normal glycated hemoglobin (HbA1c) level (5.2%; 33 mmol/mol). However, oral glucose tolerance testing (OGTT) showed elevated 2-hour glucose level of 202 mg/dL (11.2 mmol/L), with insulin greater than 1000 μU/mL (6944 pmol/L). High-density lipoprotein (HDL) cholesterol level was low (24 mg/dL [0.6 mmol/L]), and triglyceride levels were slightly elevated (161 mg/dL [1.82 mmol/L]). Hypoleptinemia was also observed (leptin: 0.94 ng/ml). Liver evaluation revealed elevated levels of transaminases (e.g., alanine aminotransferase [ALT], 168 U/L; aspartate aminotransferase [AST], 94 U/L). Vibration-controlled transient elastography (VCTE; FibroScan^®^, Echosens, Paris, France) showed steatosis (controlled attenuation parameter [CAP], 358; normal 90-248) and significantly increased liver stiffness (transient elastography, 16.4 kPa; normal <7), ruling in favor of compensated cirrhosis which was confirmed by liver biopsy.

#### 2.1.2 Management and follow-up

At age 5 years, the patient started metreleptin (0.06 mg/kg/day) with a low-fat diet and metformin (off-label use, 750 mg twice daily), with goals of improving insulin resistance and preventing further progression of liver disease. One year later, her mother noted decreased abdominal girth, near resolution of acanthosis nigricans, and decreased hunger. She had lost 4.6 kg in body weight, and linear growth had slowed. Z scores were +1.0 for height, +0.8 for weight, and +0.4 for BMI. Physical examination revealed reduced hepatomegaly, with liver edge 7 cm below the costal margin, and mild acanthosis nigricans. VCTE showed improved steatosis (CAP, 334) and fibrosis (4.7 kPa); ALT and AST levels were normal. Lipid panel testing showed HDL-cholesterol level of 29 mg/dL (0.75 mmol/L) and triglyceride level of 114 mg/dL (1.29 mmol/L). HbA1c level was 5.2% (33 mmol/mol). Oral glucose tolerance testing (OGTT) showed fasting and 2-hour glucose levels of 82 and 94 mg/dL (4.6 and 5.2 mmol/L), with corresponding insulin of 12 and 38 μU/mL (83 and 264 pmol/L). The patient’s metreleptin dose was reduced to equal 0.06 mg/kg/day based on her reduced body weight.

#### 2.1.3 Teaching points

1. This case illustrates early-onset cirrhosis that can occur in CGL2 (47). Treatment with metreleptin was followed by regression of fibrosis. Regression of even advanced fibrosis (cirrhosis) has been shown with eradication of hepatitis C. Whether metreleptin can effectively treat cirrhosis in CGL is not established (in the US, metreleptin is not approved for treatment of metabolic dysfunction-associated steatohepatitis [MASH] (56)). Nonetheless, the current multi-society lipodystrophy practice guidelines support the initiation of metreleptin in patients with GL (including CGL2), with a goal of preventing advanced hepatic fibrosis (1). Previous exploratory work involving seven patients with stage 3 fibrosis due to lipodystrophy suggests that long-term metreleptin treatment may lead to improvements in liver fibrosis (63).
2. This case also highlights the severe hyperinsulinemia that may occur in young children with CGL. This places stress on the beta cells, eventually leading to irreversible loss of beta cell function and overt diabetes (2). In this patient, although fasting glucose and HbA1c levels were normal, OGTT revealed blood glucose levels in the diabetic range at 5 years of age. In this case, metreleptin and metformin normalized both glucose and insulin levels.
3. Metreleptin can suppress the hyperphagia caused by leptin deficiency in patients with CGL, and weight loss is an expected consequence (64). In response to this weight change, the patient’s metreleptin dose was decreased to maintain a consistent dose per kilogram of body weight.
4. This patient had tall stature before metreleptin therapy, which is thought to be a result of growth promoting actions of very high insulin levels. After normalization of insulinemia with metreleptin and metformin, she experienced “catch-down” growth, thus shifting her growth parameters into the normal range.

### 2.2 Case study 2 – a case of CGL1 with mild metabolic disease

#### 2.2.1 Clinical history and diagnostic evaluation

Case 2 describes a female with CGL type 1 who is clinically managed in Europe. She was born at 39 gestational weeks to healthy consanguineous parents after an uneventful pregnancy. As a newborn, she exhibited generalized loss of subcutaneous fat, muscular hypertrophy, failure to thrive, a voracious appetite, and high serum triglyceride levels. However, hypertriglyceridemia normalized with a modified, low-fat diet. At the age of 4 years, laboratory tests revealed slightly elevated fasting insulin levels, indicative of insulin resistance, but normal HbA1c and fasting glucose levels. Notably, the patient did not present with hypertriglyceridemia at this time. Leptin levels were undetectable, and abdominal ultrasonography confirmed mild hepatic steatosis without evidence of significant fibrosis. The patient's parents reported severe hyperphagic behavior. Genetic testing revealed a homozygous nonsense variant in the *AGPAT2* gene.

#### 2.2.2 Management and follow-up

Management involved lifestyle modifications and dietary counseling to ensure a balanced diet. However, these measures did not normalize insulin sensitivity and liver steatosis. Therefore, metreleptin treatment was initiated at age 10 years (0.06 mg/kg/day). Following 9 months of treatment, insulin levels normalized, and abdominal ultrasonography showed no signs of hepatic steatosis. Moreover, hyperphagic behavior was mitigated with metreleptin. Regular follow-up visits every 6 months in a specialized center were recommended to ensure multidisciplinary care and close monitoring of her metabolic status and liver function. This monitoring approach was also advised for her male sibling who showed similar features after birth, bearing the same variant in the *AGPAT2* gene.

#### 2.2.3 Teaching points

1. Early intervention and multidisciplinary care in specialized centers are important for managing CGL1 effectively and improving long-term outcomes. Proactive management strategies may improve metabolic control and health-related quality of life in patients with CGL1 throughout their lives (52).
2. Females affected by CGL1 tend to exhibit a more pronounced and earlier onset metabolic phenotype compared with males (47). Therefore, the timing of initiation of disease-specific treatments such as metreleptin should be individualized and may be later in males compared to females. The differential effect of sex on the phenotypic expression of lipodystrophy remains incompletely understood; however, sex-based variations in adipose tissue biology and metabolism are likely contributing factors (47).

### 2.3 Case study 3 – a case of CGL4 with distinct clinical features

#### 2.3.1 Clinical history and diagnostic evaluation

Case 3 is a 6-year-old Middle Eastern male who was referred for evaluation of dysmorphic features and hepatosplenomegaly. He was born at term following an uneventful pregnancy. At 3 weeks of age, the patient developed projectile vomiting and was diagnosed with pyloric stenosis, for which he underwent surgery in a local hospital. Following recovery from pyloric stenosis, his appetite improved, and he later became hyperphagic due to leptin deficiency. The patient had a mild delay in meeting motor milestones. His parents are consanguineous and he has two healthy siblings. Upon examination, he had coarse facial features and marked fat loss from the face, extremities, palms, and soles, giving him an apparent muscularity with prominent veins. His skin was normal with no acanthosis nigricans. His weight was at the third percentile. His abdomen was distended, and he had hepatosplenomegaly, with spleen and liver palpable 4 cm below the costal margin. Neuromuscular examination revealed normal muscle strength, normal reflexes, and normal joint range of motion. Painless muscle mounding was easily elicited by finger percussion or reflex hammer on several muscles over the thenar, forearm, arm, and thigh regions.

#### 2.3.2 Management and follow-up

Laboratory investigations showed AST level at 127 U/L and ALT level at 100 U/L. His creatine kinase (CK) level was 2903 U/L (normal range, 39–308 U/L). His HbA1c level was 4.5% (26 mmol/mol), and his OGTT results were normal. Electrocardiogram (ECG) findings were also normal. His lipid profile showed triglyceride levels at 257 mg/dL (2.9 mmol/L) and serum leptin levels were undetectable. A cervical spine X-ray revealed atlantoaxial instability. Genetic workup identified a homozygous pathogenic frameshift variant c.160del (p.Val54Cysfs2) in the *PTRF/CAVIN1* gene. Metreleptin was initiated (0.06 mg/kg/day; with a low-fat diet) to treat the metabolic complications of leptin deficiency in this patient.

#### 2.3.3 Teaching points

1. CGL4 is characterized by distinct clinical features in addition to a near total lack of adipose tissue, low serum leptin levels, and a tendency to develop metabolic abnormalities during early years of life (65).
2. Myopathy is a major feature of CGL4. In patients presenting with generalized fat loss, a high serum CK level should trigger genetic testing for CGL4. Neuromuscular issues in CGL4 can hinder motor development in young children and may lead to long-term disabilities. Atlantoaxial instability is a common abnormality in CGL4 cases and can be identified with proper screening (65).
3. Patients with CGL are typically hyperphagic due to absolute leptin deficiency. However, in CGL4, gastrointestinal dysmotility and pyloric stenosis may also occur early, and these additional gastrointestinal complications can contribute to alterations in appetite (65). Although the precise mechanism of gastrointestinal disease in CGL4 is not fully understood, myopathy seems to contribute significantly to its development (65).
4. Cardiac arrhythmias including exercise-induced catecholaminergic polymorphic ventricular tachycardia, are a significant and potentially life-threatening comorbidity in CGL4 (65). Normal baseline findings on ECG do not exclude the risk of ventricular arrhythmias. To identify these arrhythmias, exercise testing may be necessary, but this can be challenging in young children who might only be able to undergo such tests when they are older.
5. In early childhood, the metabolic impact of CGL4 may appear less severe, but the condition can rapidly progress once metabolic abnormalities develop. The onset of lipid abnormalities and liver disease in CGL4 generally follows a similar pattern to other major forms of CGL, although diabetes tends to emerge at a slightly later stage (65). While this patient was not diabetic at the time of metreleptin initiation, he already exhibited lipid abnormalities and liver disease. A recent study from the US National Institute of Health (NIH) comparing patients with GL treated earlier versus later in their disease course found that those who initiated metreleptin before the onset of severe metabolic complications had better long-term control of diabetes, proteinuria, and hypertriglyceridemia, as well as less severe progression of liver fibrosis (66).

### 2.4 Case study 4 – a complicated case of AGL with autoimmune liver disease and metabolic abnormalities

#### 2.4.1 Clinical history and diagnostic evaluation

Case 4 is adolescent female who was referred to an endocrinology clinic in Europe for assessment of AGL associated with severe autoimmune liver disease, liver fibrosis and portal hypertension. She first developed acute autoimmune hepatitis at the age of 4 years and was treated with immunosuppressant therapies, including prednisolone and azathioprine. Coincident with the development of hepatitis, she had progressive generalized loss of all subcutaneous fat over a period of approximately 6 months. At age 13 years, her liver disease was stable on a low dose of prednisolone (2.5 mg on alternate days), but fibrosis and MASH were evident on a recent biopsy. She developed diabetes mellitus at the age of 10 years and was treated with insulin, given as multiple daily injections totaling 0.9 units per kg per day. She struggled with dietary adherence. On examination, she had generalized absence of subcutaneous fat, moderate acanthosis nigricans (neck and axillae), and a smooth liver edge was palpable 3 cm below the right costal margin. Low leptin levels were also detected (leptin, <0.5 ng/ml).

#### 2.4.2 Management and follow-up

In view of the coexistence of diabetes mellitus and generalized lipodystrophy, the decision was made to start treatment with metreleptin at a dose of 0.06 mg/kg/day, in addition to low-fat diet, with the aim of mitigating ongoing liver insult secondary to lipodystrophy. Improvements in metabolic parameters were observed 6 months post-metreleptin initiation. These included reductions from baseline on fasting glucose (from 227 mg/dL [12.6 mmol/L] to 132 mg/dL [7.3 mmol/L]), HbA1c (from 6.9% to 5.7% [from 52 mmol/mol to 39 mmol/mol]), fasting insulin (from 173 µU/mL [1201 pmol/L] to 94 µU/mL [653 pmol/L]), triglycerides (from 434 mg/dL [4.9 mmol/L] to 177mg/dL [2.0 mmol/L]) and ALT (65 U/L to 23 U/L). Treatment was well tolerated, with no adverse effects.

#### 2.4.3 Teaching points

1. The metabolic sequelae of AGL are comparable to those observed in CGL (1).
2. Although the primary liver insult may be autoimmune, the co-existence of lipodystrophy may result in ongoing hepatic damage secondary to fatty infiltration.
3. Management strategies in AGL are similar to those used in CGL. Adherence to diet is crucial, and metreleptin therapy may improve metabolic complications (1).

### 2.5 Case study 5 – the use of standard therapies in a patient with a delayed diagnosis of FPLD

#### 2.5.1 Clinical history and diagnostic evaluation

Case 5 is a 29-year-old female who was referred to a European clinic for high triglyceride levels. The patient was diagnosed with hypertriglyceridemia at 15 years of age (>1000 mg/dL [>11.3 mmol/L]) and was treated with omega-3 fatty acids. Hypertriglyceridemia was diagnosed while managing severe acanthosis nigricans. Development of Cushingoid facial features also occurred. Insulin levels and homeostatic model assessment for insulin resistance were high; however, no diabetes was detected. She was diagnosed with PCOS at 17 years of age due to oligomenorrhea and hirsutism and treated with metformin. At age 28, the patient initiated oral estrogen/progesterone due to amenorrhea for over 12 months. Soon thereafter, she had an episode of moderate-severe acute pancreatitis associated with splenic artery thrombosis. Lifestyle risk factors for pancreatitis (e.g., alcohol consumption) were excluded. During the event, highly elevated triglyceride levels were detected (5400 mg/dL [61 mmol/L]), and plasma apheresis was performed. Diabetes was also diagnosed at that time (HbA1c, 7.5% [59 mmol/mol]). She started metformin (500 mg), fibrate, and omega-3 fatty acids (2000 mg). Her family history was positive for high triglyceride levels (>2000 mg/dL [>22.6 mmol/L] in both her mother and her maternal uncle) and negative for pancreatitis. As the patient expressed a desire to start a family, metreleptin therapy was not initiated in this patient (as per the European prescribing information, metreleptin is not recommended during pregnancy while female patients of childbearing potential are advised to use adequate contraception, if necessary, during metreleptin treatment) (57). At age 29, her physical measurements were normal (weight, 68 kg; height, 173 cm; BMI, 22.7; waist circumference, 84 cm). Her physical appearance suggested FPLD2 with Cushingoid features, acanthosis nigricans, hirsutism, loss of adipose tissue mainly in the legs, and phlebomegaly. An abdominal computed tomography (CT) scan revealed liver steatosis with hepatomegaly, as well as suspected abdominal mesenteric lipomatosis. Low-moderate fibrosis was detected on VCTE. Kidney function was normal, but microalbuminuria was present. Reduced levels of leptin (3.5 ng/ml) and adiponectin (0.9 µg/ml) were noted. Genetic analysis revealed a pathogenic heterozygous variant in exon 8 of the *LMNA* gene (c.1145G>A) (67).

#### 2.5.2 Management and follow-up

Estrogen/progesterone was stopped. A low-fat and low-sugar diet was started. Fibrates, increased omega-3 fatty acids (4000 mg/day) and metformin (1000 mg/day) were maintained. Treatment with dapaglifozin (10 mg/day), a statin, and a medium-chain-triglyceride oil resulted in improvement of diabetes, lipid profile, and appearance of menstrual cycles.

#### 2.5.3 Teaching points

1. The phenotypic onset of FPLD2 typically becomes evident during puberty. Physical manifestations (e.g., Cushingoid appearance, acanthosis nigricans, hirsutism), PCOS, and metabolic abnormalities are usually among the first clinical features to become apparent (1–3).
2. Partial lipodystrophy may be suspected in patients presenting with Cushingoid features or severe acanthosis nigricans (1). Once a diagnosis of Cushing syndrome has been excluded, a diagnosis of partial lipodystrophy should be suspected and further investigated.
3. FPLD2 should be part of the differential diagnosis of severe dyslipidemia.
4. Protracted diagnosis of FPLD can delay initiation of appropriate medical intervention, predisposing patients to the risk of organ system complications (52, 62). This is particularly important during the transitionary period from adolescence to adulthood when metabolic complications typically develop and when age-appropriate treatment strategies directed towards chronic disease management need to be optimized.
5. If patients with FPLD and PCOS are misdiagnosed as simple PCOS, treatment with oral estrogen/progesterone can increase the risk of organ system complications. This is particularly relevant in patients with severe hypertriglyceridemia which can predispose to pancreatitis.

### 2.6 Case study 6 – a case of generalized lipodystrophy-associated progeroid syndrome

#### 2.6.1 Clinical history and diagnostic evaluation

Case 6 represents a 12-year-old Caucasian female who was referred to a clinic in the North America with generalized loss of adipose tissue that was particularly evident in her face and extremities. Physical examination revealed micrognathia, pinched nose, low-set ears, atrophic skin with mottled hyperpigmentation, and mild acanthosis nigricans. Additionally, she exhibited joint contractures of the phalangeal joints, wrists, and ankles, and hammer toes. Her medical history indicated a failure to thrive since early infancy, with the loss of subcutaneous fat becoming evident around the age of 6. She also developed mottled skin hyperpigmentation in the neck, axillae, and groin. Hepatomegaly was noted at 9 years of age. Notably, there was no known family history of lipodystrophy. At 10 years of age, the patient was diagnosed with diabetes (HbA1c, 9.5% [80 mmol/mol]), hypertriglyceridemia (750 mg/dL [8.5 mmol/L]), and elevated serum AST and ALT levels (78 and 50 IU/L, respectively). Her serum leptin level was low at 0.4 ng/ml. Genetic testing showed a *de novo* heterozygous pathogenic c.29C>T variant in the *LMNA* gene, which translates to a p.Thr10Ile amino acid substitution in the lamin A/C protein. Further investigations revealed hepatosplenomegaly with hepatic steatosis on abdominal ultrasound, which was confirmed by biopsy to be MASH without fibrosis. Echocardiogram showed valvular abnormalities.

#### 2.6.2 Management and follow-up

Insulin was initiated at 10 years of age, and metreleptin (in addition to a low-fat diet) was added at 11 years of age (0.06 mg/kg/day). This combined therapy led to improved fasting serum triglyceride levels to 150 mg/dL (1.69 mmol/L) and HbA1c level to 8% (64 mmol/mol) by 12 years of age.

#### 2.6.3 Teaching points

1. GL-associated progeroid syndrome (GL-APS) is associated with a specific pathogenic variant of *LMNA* gene at position 10 (p.T10I). This variant is usually *de novo*, but inherited variants have been documented. In GL-APS, adipose tissue is normal at birth and is lost during childhood. However, the dysmorphic features point to a genetic condition rather an acquired form of lipodystrophy (68).

### 2.7 Case study 7 – a case of CGL1 with bone cyst abnormalities

#### 2.7.1 Clinical history and diagnostic evaluation

Case 7 is a 14-year-old Middle Eastern female with CGL1 (due to a homozygous variant in the *AGPAT2* gene). Her family history includes consanguineous parents. The patient was clinically diagnosed with CGL at age of 3 years; however, clinic visits were infrequent. At age 14 years, she was admitted to a local hospital with an elbow fracture after falling from a bicycle. On physical examination she was noted to have prominent musculature, abdominal distension with mild hepatomegaly, and she had not yet started menstruation. Growth charts showed that she had experienced failure to thrive, with a weight standard deviation score consistently below –2 in past visits, while her height remained within normal limits for her age.

#### 2.7.2 Management and follow-up

Laboratory evaluation showed mild elevation of ALT level (44 IU/L) with a normal lipid profile and HbA1c of 5.5% (37 mmol/mol). An X-ray of the fractured elbow showed a bone cyst at the fracture site, and additional smaller bone cysts were detected in the extremities. Ultrasonography showed a slightly enlarged liver. Her ECG showed no arrythmias, and her echocardiogram findings were normal.

#### 2.7.3 Teaching points

1. This case highlights the presence of bone cysts in CGL (50). Although bone cysts are recognized in CGL1, fractures are not well-documented in this CGL subtype. Bones cysts are not frequently observed in other types of CGL (69).
2. Identifying bone cysts is important, as patients with CGL and bone lesions should take precautions to prevent trauma and avoid contact sports to reduce the risk of fractures (1).
3. Close monitoring for metabolic complications is recommended, and metreleptin may be considered upon diagnosis of CGL (1).

### 2.8 Case study 8 – a case of progressive encephalopathy with generalized lipodystrophy (PELD)

#### 2.8.1 Clinical history and diagnostic evaluation

Case 8 is a 5-year-old male (managed in Europe) with a rare phenotype of frequent severe seizures followed by near-complete adipose tissue loss (previously reported in (70)). The patient was born at term after an uncomplicated pregnancy to non-consanguineous parents. Within the first month after birth, the patient presented with generalized hypotonia. At 12 months of age, he developed tonic seizures that occurred monthly, which were controlled with valproic acid. At 2 years of age, his height was normal, with a low BMI and almost complete lack of subcutaneous adipose tissue. At initial evaluation, the patient presented with hypertriglyceridemia, elevated liver transaminases, and hepatic steatosis, and was prescribed a low-fat diet. He also had a psychomotor delay. In the ensuing years, seizure frequency increased accompanied by myoclonic phenomena that were resistant to pharmacologic therapy. Genetic testing revealed a homozygous c.(1076dupC)/p.(Glu360*) *BSCL2* variant. At 4 years of age, his hypertriglyceridemia moderately improved with low-fat diet; further evaluations revealed hepatomegaly with steatosis and arrhythmia was found on ECG. While he was able to walk, he had frequent falls due to myoclonic seizures. Moreover, he had severe intellectual disability, and electroencephalography (EEG) findings indicated worsening of the seizures.

#### 2.8.2 Management and follow-up

The patient required several hospitalizations for prolonged myoclonic seizures. Therefore, a vagal nerve stimulator was implanted. Perampanel was initiated after seizures reappeared. Daily seizures (up to 60 seizures episodes per day) persisted, without status epilepticus. Ten months later, at 5 years of age, he was hospitalized with new episodes of prolonged seizures with mixed tonic and myoclonic components. Subcutaneous metreleptin 0.06 mg/kg/day was initiated and was well tolerated. After 2 months of therapy, his overall lipid profile improved; neurological status was stable without prolonged seizures. Serum leptin level (0.4 ng/mL) was normal-to-low for his BMI (0.25-3.20 ng/mL) before metreleptin. After 1 year of treatment with metreleptin, liver function improved. The patient’s height velocity was 8.1 cm/year, and his BMI was below the third percentile. In the subsequent 2 years, he did not experience any prolonged myoclonic epileptic seizures, and the frequency of hospitalization decreased but he still experienced 2 to 3 seizures per month, and daily myoclonic seizures involving mainly the perioral muscles.

#### 2.8.3 Teaching points

1. This case provides evidence that certain specific variants in *BSCL2* are associated with systemic effects not limited to generalized absence of adipose tissue. It is unknown whether myoclonic epilepsy is related to altered *BSLC2* expression in the brain; however, the improvement in myoclonic seizures following the introduction of metreleptin may suggest a contributory role for leptin deficiency in neurodegeneration (70).

### 2.9 Case study 9 – a case of CGL2 with extreme hyperphagia

#### 2.9.1 Clinical history and diagnostic evaluation

Case 9 is a 10-year-old female (managed in South America) who was diagnosed with CGL2 at age 8 months. She had a sister with the same diagnosis. She exhibited generalized loss of subcutaneous fat and muscular hypertrophy since birth, failure to thrive, voracious appetite and high serum triglycerides (407 mg/dL [4.6 mmol/L]). She had hepatomegaly without clinically significant fibrosis, and slightly elevated fasting insulin without diabetes. After starting a low-carbohydrate and low-fat diet, her hyperphagia led to socially dysfunctional food-seeking behaviors, including stealing food at school, and hitting peers to obtain food. Her most recent BMI measure was 14.1 (Z-score, -1.07) and blood pressure was at 102/64 mmHg. Echocardiogram and abdominal ultrasound were normal. Genetic testing revealed a pathogenic nonsense variant in the *BSCL2* gene, c414, deletion (p.Cys138Ter) in exon 3.

#### 2.9.2 Management and follow-up

Management included lifestyle modifications and dietary counselling to ensure a balanced diet. Aripiprazole was initiated to help control her aggressive behavior at school and at home.

#### 2.9.3 Teaching points

1. This case highlights a patient with CGL and hypertriglyceridemia, where voracious hunger was the predominant issue, leading to significant social dysfunction and aggressive behavior. Furthermore, this case underscores the impact of uncontrolled hunger on social interactions and behavior from early childhood (1–3).

### 2.10 Case study 10 – management of CGL in a patient with allergy to metreleptin

#### 2.10.1 Clinical history and diagnostic evaluation

Case 10 is a female patient (managed in Europe) who was diagnosed with CGL1 at 2 months of age. Diagnosis was supported by a generalized lack of adipose tissue, morphological features characteristic of CGL, insulin-resistant diabetes, hypertriglyceridemia, and elevated liver enzymes. Metabolic abnormalities resolved within few months following the initiation of a low-fat diet and medium chain triglycerides supplementation without need for insulin or other medications. Insulin resistance reappeared at age 11 years (isolated elevated fasting insulinemia), and she developed clinical diabetes at age 13.5 years with a corresponding increase in HbA1c levels from 6% to 11% (from 42 mmol/mol to 97 mmol/mol) within a 4-month period. Lipids levels and liver enzymes remained in the normal range.

#### 2.10.2 Management and follow-up

Insulin was initiated shortly after the diagnosis of diabetes followed by metformin. Metreleptin was initiated 6 months later (at age 14 years) but the patient developed allergic reactions (skin redness at injection sites and itchy throat) after 1 month of treatment and was stopped. HbA1c was 10.5% (91 mmol/mol) despite increased insulin doses. Dapagliflozin (10 mg/daily) and semaglutide (0.5 mg/week) were later introduced after which total daily insulin requirements decreased from 1.5 to 0.6 units/kg/day and HbA1c level reduced to 6.4% (46 mmol/mol). This treatment regimen was maintained for 5 years up to the present day. However, during early adulthood, the patient developed hypertriglyceridemia and persistent microalbuminuria that were controlled with fenofibrate and enalapril, respectively.

#### 2.10.3 Teaching points

1. In CGL, metabolic complications can occur early in life. Treatment of metabolic complications should be initiated as soon as they are detected (e.g., at the time insulin resistance was detected) to limit the progression of disease.
2. In situations where treatment with metreleptin is not possible (e.g., unavailable in the country, contraindicated), glucose-lowering agents such as sodium-glucose cotransporter-2 (SGLT2) inhibitors or glucagon-like peptide-1 receptor agonists (GLP-1 RAs), may help improve glycemic control in patients with partial lipodystrophy (71–73). Recent case series and retrospective analyses have also reported on the use of tirzepatide, a dual glucose-dependent insulinotropic polypeptide (GIP)/GLP-1 receptor agonist, in adult patients with lipodystrophy presenting with diabetes (74, 75). However, the efficacy and safety of these agents have not yet been established in patients with lipodystrophy in clinical trials.

## 3 Discussion

Here, we present a series of 10 pediatric patients with lipodystrophy (comprising six patients with CGL, and one patient each with AGL, FPLD, PELD, and GL-APS) and describe the clinical approach used by us in these individuals.

### 3.1 The heterogeneous clinical presentation of lipodystrophy syndromes in pediatric patients

Our cases illustrate the broad phenotypic spectrum associated with lipodystrophy syndromes. The predominant phenotypic feature of lipodystrophy, adipose tissue loss, usually presents at birth in patients with CGL and can be identified through careful physical examination. Patients with AGL and ultra-rare subtypes, such as GL-APS, may experience adipose tissue loss later in childhood. In patients with FPLD, loss of adipose tissue may start during childhood but usually becomes noticeable around puberty and adolescence (1–3).

The ages at which adipose tissue loss was detected in our patients are consistent with previous natural history studies of lipodystrophy; for example, data from Spain reported the mean age at phenotypic onset of GL at 4.7 years and 16.5 years in PL (45). Several physical features associated with lipodystrophy syndromes (e.g., acanthosis nigricans, muscular hypertrophy, and abdominal distension due to hepatomegaly) were also evident in many of our patients. In contrast, the single patient with FPLD2 (Case 5) did not receive a diagnosis of partial lipodystrophy until adulthood when reduced adiposity became apparent. These findings support physical appearance as a main driver of diagnosis of lipodystrophy syndromes, while family history (including parental consanguinity) supported diagnosis in some of our patients with a genetic etiology (1, 44, 47, 52).

Our patients with CGL shared characteristics that phenotypically define this lipodystrophy subtype. However, other clinical features associated with distinct CGL subtypes were observed in some individuals. These include musculoskeletal abnormalities and elevated CK levels in the patient with CGL4 (Case 3), while the early onset progression of steatosis to cirrhosis in a patient with CGL2 (Case 1) lends support for the rapid development of liver disease in this CGL subtype (30, 50, 65). The etiology of complications associated with the different CGL subtypes is not fully understood; however, some insights have been provided by case reports and murine studies. For example, muscular hypertrophy and the development of thick muscularis mucosa has been documented in patients with CGL4 presenting with pyloric stenosis (22, 23, 65). Studies in mice models further reveal that *Cavin1^-/-^* mice develop loss of cardiomyocyte caveolae accompanied by cardiomyocyte hypertrophy, cardiac fibrosis and ECG irregularities, which may account for the cardiac complications in CGL4 (although cardiac complications were not detected in Case 3) (76, 77). Similarly, patients with CGL2 generally have the most severe metabolic disease (including elevated liver enzymes) among the different CGL subtypes and this may contribute to the earlier hepatic pathology observed for this subtype. It is interesting to note that murine studies have shown that liver-specific deficiency of seipin did not lead to hepatosteatosis, suggesting that the metabolic dysfunction associated with CGL2 is predominantly driven by the function of *BSCL2* at the adipose tissue level (32, 50, 78).

We also describe the clinical presentation of two rare variants in the *LMNA* and *BSCL2* genes (Cases 6 and 8). Variants in *LMNA* are generally associated with the FPLD2 phenotype; however, Case 6, who was heterozygous for the *LMNA* p.T10I variant, developed GL-APS. Consistent with earlier phenotypic descriptions of this genotype, Case 6 presented with failure to thrive in infancy and developed severe metabolic complications by late childhood (68). The *LMNA* p.T10I variant results in the expression of mutant lamin A protein that causes detachment of chromatin to nuclear lamina and possible impaired cellular division (79). Similarly, Case 8 demonstrates how rare variants in *BSCL2* can manifest as PELD (or Celia’s encephalopathy, https://www.omim.org/entry/615924) — a genetic disorder that presents with generalized lipodystrophy and severe progressive neurodegeneration from an early age (32, 70). PELD is typically associated with variants that cause exon 7 skipping, notably the c.985C>T variant, which disrupts normal splicing and results in a truncated seipin isoform (Celia-seipin) lacking the second transmembrane domain. This misfolded protein forms macroaggregates, triggering endoplasmic reticulum stress, activation of the unfolded protein response, and neuronal apoptosis, ultimately leading to early-onset progressive myoclonic epilepsy and severe neurodegeneration. Although histopathological confirmation was lacking in Case 8, molecular similarities with previously reported *BSCL2* variants (e.g., c.974dupG) support a shared pathogenic mechanism with PELD (80–82). The clinical evidence presented for Case 8 also suggests that leptin deficiency may contribute to neurodegeneration in PELD; however, further work is needed to explore this (70).

Finally, while males and females with lipodystrophy exhibit similar patterns of fat loss, sex effects on the expression of the lipodystrophy phenotype have been reported in some subgroups. For example, in CGL, females generally display more severe comorbidities than males (illustrated here by Cases 1 and 2), possible due to sex-based differences in adipose tissue physiology. Females generally have a greater proportion of subcutaneous adipose tissue and exhibit higher circulating leptin levels compared to males. Subcutaneous adipose tissue in women expresses higher levels of estrogen receptors, which promote adipocyte differentiation upon estrogen binding. Furthermore, sexual dimorphism in adipose tissue distribution is well established, with females preferentially accumulating adipose tissue in the gluteofemoral region, particularly during periods of positive energy balance. These findings highlight the critical role of adipose tissue in female metabolic regulation and suggest that adipose tissue depletion may lead to a greater degree of metabolic abnormality in women than in men (47, 83–85).

### 3.2 The importance of early diagnosis in the holistic management of lipodystrophy

Our cases highlight the importance of early diagnosis in the holistic management of lipodystrophy syndromes. Due to their rarity, the awareness of lipodystrophy syndromes among the general medical community is often limited, leading to diagnostic delays (1, 32, 52). Recent analysis from France estimated a median diagnostic delay of 10.7 years for CGL (n=18) and 10.5 years for FPLD2 (n=67) (62). Similarly, assessment of a Turkish GL cohort (N=72) showed that the average delay in diagnosis was ~4.3 years (52 months) (47).

Protracted diagnosis times can delay medical intervention and expose patients to the risk of developing severe comorbidities, as evidenced by our patient with FPLD2 (Case 5). This observation is further supported by data from an international chart review study involving 230 patients, whereby the diagnosis of GL and PL occurred, respectively, 3.1 years and 9.0 years after the first complications of lipodystrophy were detected (43). It has been proposed that coordinated care pathways that support the referral of suspected lipodystrophy cases to specialized centers could help expedite diagnosis and initiation of appropriate management. This is particularly important for pediatric patients with lipodystrophy syndromes as early intervention may help optimize long-term clinical outcomes (52, 62).

The potential of digital technologies in aiding diagnosis of lipodystrophy syndromes has been demonstrated. Examples include the LipoDDx mobile application, which has an effectiveness of 80% in identifying lipodystrophy subtypes from the medical records of individuals with generalized or partial adipose tissue loss (86). Similarly, a pilot study demonstrated that the deep learning algorithm of the DEEPLIPO tool distinguished patients with CGL from non-lipodystrophic individuals with athletic build and individuals with malnutrition with mean accuracy, sensitivity, and specificity of 91% each (87). Furthermore, the recently developed lipodystrophy disease severity score (LDS) provides a tool that holistically captures the diverse clinical features and manifestation of lipodystrophy syndromes into a numerical score that may be used to monitor disease progression and the clinical effect of treatment (88). Additional work is needed to establish how these digital technologies may support diagnosis of suspected lipodystrophy cases in routine clinical settings (86, 87).

### 3.3 Considerations for the clinical management of lipodystrophy syndromes in pediatric patients

Clinicians managing pediatric patients with lipodystrophy syndromes face certain challenges that may be encountered less frequently in adult patients. For instance, dietary restriction and control of hyperphagia may be difficult to manage in young leptin-deficient patients, particularly during periods of active growth (1). This is supported by hyperphagia in Case 9 that resulted in severe food seeking and anti-social behavior. Children may not be able to sufficiently describe their symptoms, hindering diagnosis and monitoring clinical outcomes while on treatment. In this regard, management strategies for pediatric patients may differ from those used in adult patients.

Here, we describe the clinical management strategies used by us for nine of our 10 patients (seven with GL, one with GL-APS and one with FPLD). A low-carbohydrate, low-fat diet was initiated in all nine patients following the onset of lipodystrophy. This is aligned with the current multi-society guidelines for lipodystrophy which recommend a diet consisting of 50–60% carbohydrate, 20–30% fat, and approximately 20% protein; however, these recommendations are based on expert opinion and diets should be individualized (1). Metreleptin is recommended as the first line treatment by the current multi-society practice guideline to treat complications of leptin deficiency in GL (1); however, some of our patients were initially treated with widely available glucose- and lipid-lowering agents. Notably, the limited effectiveness of these agents in some of these patients led to the use of metreleptin. The findings from a NIH clinical study involving 53 patients with lipodystrophy syndromes aged <18 years support the use of metreleptin in the pediatric population. In that study, significant mean reductions from baseline were observed at month 12 for fasting glucose levels (from 176 to 121 mg/dL [from 9.8 to 6.7 mmol/L]) and HbA1c (from 8.3% to 6.5% [from 67 mmol/mol to 48 mmol/mol]), with concomitant reductions in the number of patients requiring insulin. Significant median reductions from baseline were also observed at month 12 for triglycerides (from 374 mg/dL to 189 mg/dL [from 4.2 mmol/L to 2.1 mmol/L]), ALT (from 73 to 41 U/L) and AST (from 51 to 26 U/L). These metabolic improvements were sustained over mean duration time of 61 months (61).

Other research has demonstrated broad clinical effects of metreleptin in lipodystrophy, supporting a pleiotropic role of leptin. These include amelioration of hyperphagia, hepatic, cardiovascular and renal complications, normalization of gonadotropin secretion and menstrual cycles (in females), and improved quality-of-life and survival parameters. Of note, these studies primarily involved adult patients with lipodystrophy (49, 64, 89–96).

Metreleptin is generally well-tolerated in the pediatric lipodystrophy population (61). The most common safety events in this patient group were decreased appetite and/or weight loss, hypoglycemia and fatigue (61). In the US, metreleptin is available only through a restricted program and the prescribing information carries a black box warning for the risk of anti-metreleptin antibodies with neutralizing activity and the risk of lymphoma (56). These warnings are also included in the metreleptin prescribing information from Europe and metreleptin is subject to additional monitoring in this region. Other adverse reactions documented with metreleptin include, but are not limited to, hypersensitivity reactions (as reported for Case 10), acute pancreatitis following discontinuation of metreleptin, and hypoglycemia with concomitant use of insulin and other glucose-lowering agents (56–58).

The use of metreleptin, a daily injectable treatment, may be challenging in young patients. The near-complete absence of subcutaneous adipose tissue may hamper injection technique (56–58). Administering injections to an active child can be difficult and may be source of anxiety for patients and their guardians. Weight loss while on metreleptin (often driven by reductions in hyperphagia) can be distressing for some young patients, especially those who already have a thin physical appearance due to reduced adiposity (97). Furthermore, some young patients may require metreleptin with other concomitant therapies, which may be burdensome and lead to poor treatment adherence (98).

Two of our patients, one with CGL (Case 10) and one with FPLD (Case 5), received a GLP-1 RA and/or an SGLT2 inhibitor for treatment of diabetes. The use of SGLT2 inhibitors and GLP-1 RAs in patients with lipodystrophy is largely limited to retrospective studies involving adult patients with FPLD (71, 72). Results from early studies involving these agents in patients with FPLD revealed improvements in glycemic control but no significant effects on lipid parameters (71, 72). Subsequent analysis of 76 adult patients with FPLD from the French national rare disease reference network demonstrated significant short-term (12 ± 6 months) reductions from baseline for BMI, HbA1c, and triglycerides without significant changes in the use of other glucose- or and lipid-lowering medications (73). Effects on BMI, HbA1c, and triglycerides relative to baseline persisted in the long term (≥ 18 months). The most frequently reported adverse events in this study were gastrointestinal, affecting 34% of patients (73). More recently, two publications reported on the use of tirzepatide for metabolic improvement in adult patients with lipodystrophy (74, 75). Reductions in BMI, HbA1c, triglycerides and insulin requirements were observed in 14 patients with FPLD who were prescribed tirzepatide for the treatment of diabetes (median treatment period of 9 months). In the same study, tirzepatide was used in a single patient atypical PL and two patients with AGL (75). Elsewhere, two patients with CGL received tirzepatide for the treatment of diabetes (74). Tirzepatide was discontinued in one patient with CGL due to a gastrointestinal adverse event, while, in the other patient, improvements in glycemic parameters were observed at the maximum dose of 15mg/week although the observation was limited to three weeks (74). These data suggest that GLP-1 RAs or dual GIP/GLP-1 receptor agonists may offer metabolic improvement in some patients with lipodystrophy, including scenarios where daily injections are not tolerated or in geographical regions where metreleptin is not available. However, it is important to note that SGLT2 inhibitors, GLP-1 RAs and tirzepatide have not been systemically evaluated in patients with lipodystrophy. Furthermore, tirzepatide is not indicated for use in pediatric patients (99, 100).

Mibavademab (previously called REGN4461), a leptin receptor agonist, is also in development for the treatment of GL (101). Results from a Phase 2 study involving 16 patients with GL (aged ≥ 12 years) did not reveal any clinically meaningful differences for the primary efficacy endpoints (HbA1c and fasting triglycerides) after 8 weeks on low dose mibavademab compared with placebo (102). However, exploratory pooled analyses conducted after 28 weeks of mibavademab administration, showed that serum drug concentrations were significantly higher than observed at Week 8 with clinically significant reductions from baseline in HbA1c (mean, -1.9%) and in triglycerides (median, -49.3%). Treatment was generally well-tolerated with no serious adverse events related to mibavademab administration (102). While larger clinical trials are needed, mibavademab may represent a future therapeutic option for patients with GL.

## 4 Conclusion

Pediatric patients with lipodystrophy syndromes present a challenge to clinicians with limited expertise of this rare disease and management strategies are likely to differ from those typically used in adult patients. In this manuscript, we have presented a series of pediatric patients with lipodystrophy syndromes and describe our clinical approach to them. We envisage that this information may help clinical teams seeking to promptly diagnose and holistically manage patients with this rare disease and support their young patients with lipodystrophy syndromes into adulthood.
