# Supplementary material for: The clinical approach to child and adolescent patients with lipodystrophy: a series of international case discussions
Source: Front Endocrinol (Lausanne). 2025 Aug 6;16:1597053. doi: 10.3389/fendo.2025.1597053 (PMC12366100; doi:10.3389/fendo.2025.1597053)
Supplement: Supplementary file 1 [file Image1.pdf]

The clinical approach to child and adolescent patients with lipodystrophy: a series of international case discussions

Case summaries (see full manuscript for further details)

Case 1 6y F

CGL2 with early onset advanced liver disease

LD type *(gene)*
CGL2 (*BSCL2*)

Age at first signs of LD <5y

Key clinical features
- Consanguineous parents
- Hypertriglyceridemia, hyperinsulinemia
- Hyperphagia; hypoleptinemia
- Acanthosis nigricans
- Blood glucose levels in the diabetic range
- Elevated transaminase levels
- Steatosis progressing to cirrhosis

Management
- Metformin and metreleptin initiated (age 5 years, with low-fat diet)

Learnings and follow-up outcomes*
- Early-onset cirrhosis can occur in CGL2.[1]
- Severe hyperinsulinemia may also occur in young children with CGL2.
- Metreleptin led to regression of fibrosis; however, the effectiveness of metreleptin in treating cirrhosis has not been established.[2]

Case 2 11y F

CGL1 with mild metabolic disease

LD type *(gene)*
CGL1 (*AGPAT2*)

Age at first signs of LD From birth

Key clinical features
- Consanguineous parents
- Muscular hypertrophy, failure to thrive
- Hyperphagia
- Elevated TG levels in infancy
- Undetectable leptin
- Insulin resistance
- Mild hepatic steatosis

Management
- Low-fat, balanced diet
- Metreleptin initiated (age 10 years)

Learnings and follow-up outcomes*
- Females with CGL1 tend to exhibit a more pronounced and earlier onset metabolic phenotype versus males.[1]
- 9 months of metreleptin and metformin normalized insulin levels and resolved hepatic steatosis.

Case 3 6y M

CGL4 with distinct clinical features

LD type *(gene)*
CGL4 (*PTRF/CAVIN1*)

Age at first signs of LD <6y

Key clinical features
- Consanguineous parents
- Pyloric stenosis at 3 weeks of age
- Painless muscle mounding on several muscles over the thenar, forearm, arm, and thigh regions
- Undetectable leptin; hyperphagic
- Elevated transaminase, TG and CK levels
- Normal HbA1c levels and ECG profile

Management
- Metreleptin initiated (age 6 years, with low-fat diet)

Learnings and follow-up outcomes*
- Myopathy, elevated CK levels and gastrointestinal complications can occur in CGL4.[3]
- Cardiac arrhythmias are a significant comorbidity in CGL4.[3] Normal baseline findings on ECG do not exclude the risk of arrhythmias.
- Metabolic comorbidities of CGL4 may appear less severe versus other CGL types, but can progress rapidly once they develop.[3]

Case 4 13y F

AGL with autoimmune disease and metabolic abnormalities

LD type *(gene)*
AGL

Age at first signs of LD 4y

Key clinical features
- Generalized fat loss following the development and treatment of acute autoimmune hepatitis with immunosuppressant therapies (age 4 years)
- Diabetes (age 10 years)
- MASH and liver disease (age 13 years)
- Non-adherence to low-fat diet
- Moderate acanthosis nigricans
- Elevated ALT and TG

Management
- Low-fat diet
- Insulin started (age 10 years)
- Metreleptin introduced (age 13 years)

Learnings and follow-up outcomes*
- The metabolic sequelae of AGL are comparable to those in CGL.
- The co-existence of autoimmune hepatitis and lipodystrophy may result in ongoing hepatic damage.
- Metreleptin led to improvements in metabolic health and liver enzymes.

Case 5 29y F

A patient with a delayed diagnosis of FPLD

LD type *(gene)*
FPLD (*LMNA*)

Age at first signs of LD 15y

Key clinical features
- Age 15 years: hypertriglyceridemia, severe acanthosis nigricans and insulin resistance
- Age 17 years: PCOS with oligomenorrhea and hirsutism
- Age 28 years: acute pancreatitis, hypertriglyceridemia and diabetes
- Age 29 years: FPLD2 diagnosed supported by reduced adiposity in legs and abdomen, Cushingoid features, phlebomegaly and hepatic steatosis

Management
- Low-fat, low-carbohydrate diet
- Metformin (age 15 years)
- Estrogen/progesterone for amenorrhea (age 28 years)**
- Fibrates, omega-3 fatty acids, metformin (age 28 years)
- SGLT2i (for diabetes), statin and MCT oil (age 29 years)

Learnings and follow-up outcomes*
- Phenotypic onset of FPLD2 typically becomes clearly evident during puberty.[4,5]
- Delays in diagnosis of FPLD can predispose patients to the risk of organ system complications.[6]
- Metabolic improvement and appearance of menstrual cycles following a treatment regimen involving fibrates, omega-3 fatty acids, metformin, SGLT2i, a statin, and MCT oil.

Case 6 12y F

Generalized LD-associated progeroid syndrome (GL-APS)

LD type *(gene)*
GL-APS (*LMNA*)

Age at first signs of LD 6y

Key clinical features
- Failure to thrive since early infancy
- Hepatomegaly at age 9 years
- Diabetes, hypertriglyceridemia and elevated transaminases (age 10 years)
- Low leptin levels
- GL-APS confirmed by genetic testing

Management
- Insulin (age 10 years)
- Metreleptin (age 11 years, with low-fat diet)

Learnings and follow-up outcomes*
- In GL-APS, adipose tissue is normal at birth and is lost during childhood.[7]
- Dysmorphic features point to a genetic rather than acquired form of lipodystrophy.
- Metabolic improvement after 12 months of combined insulin and metreleptin.

Case 7 14y F

CGL1 with bone cysts

LD type *(gene)*
CGL1 (*AGPAT2*)

Age at first signs of LD 3y

Key clinical features
- Consanguineous parents
- Diagnosis of CGL confirmed following hospital admission due to bone fracture
- X-ray showed bone cyst at fracture site
- Normal profiles for ECG, lipids and HbA1c
- Mild elevation in ALT
- Liver slightly enlarged

Management
- Close monitoring for bone cysts and metabolic complications

Learnings and follow-up outcomes*
- Although bone cysts are recognized in CGL, fractures are not well-documented.[8]
- Identifying bone cysts in CGL can help guide strategies to prevent trauma and reduce risk of fractures.

Case 8 5y M

Progressive encephalopathy with GL (PELD)

LD type *(gene)*
PELD (*BSCL2*)

Age at first signs of LD 2y

Key clinical features
- Generalized hypotonia and frequent tonic seizures (age 1 year)
- Hypertriglyceridemia, elevated transaminases, hepatic steatosis (age 2 years)
- Liver and cardiac abnormalities (age 4 years)
- Deterioration of neurological condition (age 4 years)

Management
- Valporic acid and vagal nerve stimulator implant
- Perampanel following reappearance of seizures
- Metreleptin (age 5 years, with low-fat diet)

Learnings and follow-up outcomes*
- Specific variants in *BSCL2* may be associated with systemic effects, including neurological manifestations, beyond the generalized absence of adipose tissue.[9]
- ≥2 years metreleptin: patient experienced 2 to 3 seizures per month but no prolonged epileptic seizures leading to hospitalization.[9]

Case 9 10y F

CGL2 with extreme hyperphagia

LD type *(gene)*
CGL2 (*BSCL2*)

Age at first signs of LD From birth

Key clinical features
- Failure to thrive
- Elevated TGs
- Hepatomegaly without clinically significant fibrosis
- Slightly elevated fasting insulin without diabetes
- Severe hyperphagia leading to socially dysfunctional food-seeking behaviors (e.g., stealing food and aggressive behavior to obtain food)
- Normal ECG and abdominal ultrasound

Management
- Lifestyle modifications and dietary counseling
- Aripiprazole to help control aggressive behavior

Learnings and follow-up outcomes*
- Voracious hunger leading to significant social dysfunction and aggressive behavior is a feature of CGL that can manifest early in childhood.[4,5]

Case 10 19y F

CGL in a patient allergic to metreleptin

LD type *(gene)*
CGL1 (*AGPAT2*)

Age at first signs of LD 2mo

Key clinical features
- Insulin-resistant diabetes
- Elevated liver enzymes
- Clinical diabetes (age 13.5 years)
- Hypertriglyceridemia in early adulthood

Management
- Low-fat diet and MCT supplementation
- Metreleptin (age 14 years) but stopped due to allergic reactions
- Dapagliflozin and semaglutide later initiated to treat diabetes
- Fenofibrate (in early adulthood) to treat hypertriglyceridemia

Learnings and follow-up outcomes*
- In situations where treatment with metreleptin is not possible, glucose-lowering agents (e.g., SGLT2i or GLP-1 RAs) may help improve glucose metabolism.
- The efficacy and safety of SGLT2i or GLP-1 RAs have not been established in patients with lipodystrophy in clinical trials, nor in pediatric patients with lipodystrophy.[10-12]

***Findings are reported on a case-by-case basis; clinical presentations and outcomes may vary between patients with lipodystrophy syndromes; **later stopped due to the development of acute pancreatitis.**

AGL, acquired generalized lipodystrophy; ALT, alanine transaminase; CGL, congenital generalized lipodystrophy; CK, creatine kinase; ECG, electrocardiogram; F, female; FPLD, familial partial lipodystrophy; GLP-1 RA, glucagon-like peptide-1 receptor agonist; HbA1c, glycated hemoglobin; LD, lipodystrophy; M, male; MASH, metabolic dysfunction-associated steatohepatitis; MCT, medium-chain-triglyceride; mo, months; PCOS, polycystic ovary syndrome; SGLT2i, sodium-glucose cotransporter-2 inhibitor; TG, triglycerides; y, years

References (PubMed ID unless stated)

1. 36946378; **2.** Chiesi, MYALEPT™ US, prescribing information [available from: https://www.dailymed.nlm.nih.gov; last accessed June 2025]; **3.** 38234231; **4.** 27710244; **5.** 15028826; **6.** 38952397; **7.** 29267953; **8.** 27144933; **9.** 33099310; **10.** 35301125; **11.** 38300898; **12.** 39829337
